# Ubiquitously specific protease 4 inhibitor‐Vialinin A attenuates inflammation and fibrosis in S100‐induced hepatitis mice through Rheb/mTOR signalling

**DOI:** 10.1111/jcmm.16180

**Published:** 2020-12-09

**Authors:** Jie Xu, Dazhi Chen, Lanling Jin, Zhengkang Chen, Yulu Tu, Xiaozhe Huang, Feiben Xue, Jialu Xu, Mingzhuan Chen, Xiaodong Wang, Yongping Chen

**Affiliations:** ^1^ Department of Infectious Diseases Zhejiang Provincial Key Laboratory for Accurate Diagnosis and Treatment of Chronic Liver Diseases Wenzhou Key Laboratory of Hepatology Hepatology Institute of Wenzhou Medical University The First Affiliated Hospital of Wenzhou Medical University Wenzhou Zhejiang China; ^2^ Department of Gastroenterology The First Hospital of Peking University BeiJing China

**Keywords:** autoimmune hepatitis, mTOR, USP4, Vialinin A

## Abstract

Inflammation and fibrosis are major consequences of autoimmune hepatitis, however, the therapeutic mechanism remains to be investigated. USP4 is a deubiquitinating enzyme and plays an important role in tissue fibrosis and immune disease. Vialinin A is an extract from mushroom and is a specific USP4 inhibitor. However, there is lack of evidences that Vialinin A plays a role in autoimmune hepatitis. By employing S100‐induced autoimmune hepatitis in mice and AML12 cell line, therapeutic mechanism of Vialinin A was examined. Inflammation was documented by liver histological staining and inflammatory cytokines. Fibrosis was demonstrated by Masson, Sirius red staining and fibrotic cytokines with western blot and real‐time RT‐PCR. In experimental animal, there were increases in inflammation and fibrosis as well as USP4, and which were reduced after treatment of Vialinin A. Vialinin A also reduced Rheb and phosphorylated mTOR. Moreover, in LPS‐treated AML12 cells, LPS‐induced USP4, inflammatory and fibrotic cytokines, phosphorylated mTOR and Rheb. Specific inhibitory siRNA of USP4 reduced USP4 level and the parameters mentioned above. In conclusion, USP4 was significantly elevated in autoimmune hepatitis mice and Vialinin A reduced USP4 level and attenuate inflammation and fibrosis in the liver. The mechanism may be related to regulation of Rheb/mTOR signalling.

## 1 INTRODUCTION

Autoimmune hepatitis is chronic hepatitis with complicated causes, hepatocytes are destructed through immune‐mediated way, which mainly affects women and characterized by hypergammaglobulinanemia, circulating auto‐antibodies associated with AIH,^1^, ^2^ and interface hepatitis in liver histology. However, AIH remains a major therapeutic challenge because of its rarity and heterogeneity.^3^, ^4^ Appropriate immunosuppressive therapy may delay progress and drives the disease into remission, albeit side effects are inevitable.^5^ On the contrary, longer‐term uncontrolled disease may also cause AIH progression to fibrosis and cirrhosis.^1^ S100 is a hepatic antigen, which prepared from liver of the same species mice. Intraperitoneal injection leads to immune‐mediated hepatitis in mice. The model might allow the study of the pathogenesis of immune‐mediated hepatic failure, for instance, autoimmune hepatitis (AIH).^6^, ^7^

Ubiquitination is a reversible process and the ubiquitin moieties removed by deubiquitinating enzymes (DUBs).^8^, ^9^ Ubiquitin‐specific proteases (USPs), the largest subfamily of DUBs which divided into five families, comprise of more than 60 members in human.^10^ USP4 is a member of the USP family and closely related to tissue fibrosis progression^11^ and immune disease development.^12^ However, the role of USP4 in liver fibrosis is controversial as previous reported.^8^, ^11^, ^13^ USP4 deficiency intensifies the inflammation and fibrosis progress in the liver of hepatic I/R injury and NAFLD, While the results were reversed hepatocellular carcinoma. Therefore, the role of USP4 requires further exploration.

Vialinin A is a p‐terphenyl compound isolated from edible Chinese mushroom T. terrestris and T. vialis,^14^ and also a specific inhibitor of USP4. Vialinin A has been shown to, significantly suppresses the expression of USP4 in vitro in previous studies.^12^, ^15^, ^16^ Moreover, a research showed that Vialinin A attenuated the progression of the autoimmune disease model in an in vitro model.^12^ However, the role of Vialinin A in the in the vivo model remains unclear, as well as the beneficial effect of Vialinin A on autoimmune hepatitis. Therefore, in the current research, we demonstrate the role of USP4 in autoimmune hepatitis induced fibrosis and examine the function of Vialinin A in prevention of liver fibrosis.

## 2 MATERIALS AND METHODS

### 2.1 Antibodies and reagents

Anti‐COL‐1 (ab96723), anti‐αSMA (ab32575), anti‐TGF‐β (ab92486), anti‐FN14 (ab109365), anti‐phospho‐mTOR (ab109268), anti‐mTOR (ab2732), anti‐Ubiquitin (ab7780), anti‐GAPDH (ab181602) primary antibodies and secondary antibodies of goat anti rabbit were obtained from Abcam. Anti‐USP4 (pa5‐53820) antibodies were purchased from Thermo Fisher. Antibody of against Rheb (13879S), was obtained from CST. Vialinin A (10010519) was obtained from Cayman. LPS (L2880), ITS (I3146), Complete Freund's adjuvant (F5881) and Incomplete Freund's adjuvant (F5506) were purchased from Sigma. (R)‐MG‐132 (sc‐351846), Chloroquine diphosphate salt (sc‐205629) was obtained from Santcruz. Cycloheximide (HY‐12320) was purchased from MCE. DMEM/F‐12, FBS, amino acids (50×), β‐mercaptoethanol, penicillin and streptomycin were purchased from Gibco. PAGE precast gels, Protein A + G Agarose (P2012), normal rabbit IgG (A7016), PMSF (ST506), Phosphatase inhibitor (P1045), Cell lysis buffer (P0013) and BCA Protein Assay Kit (P0010) were purchased from Beyotime. Tissue Lysis Buffer (AR0101) was purchased from Boster. Bull serum albumin (4240gr500) was obtained from biofroxx. Glycine (G8200), SDS (S8010) and Tris (T8060) were purchased from Solarbio. Opti‐MEM, ultra‐sensitive ECL (34095), Lipofectamine 2000 Reagent were purchased from Thermo Fisher. ECL was purchased from Advansta. Dexamethasone (D137736) was obtained from Aladdin.

### 2.2 Establishment of autoimmune hepatitis model in experimental murine

**FIGURE 1:**
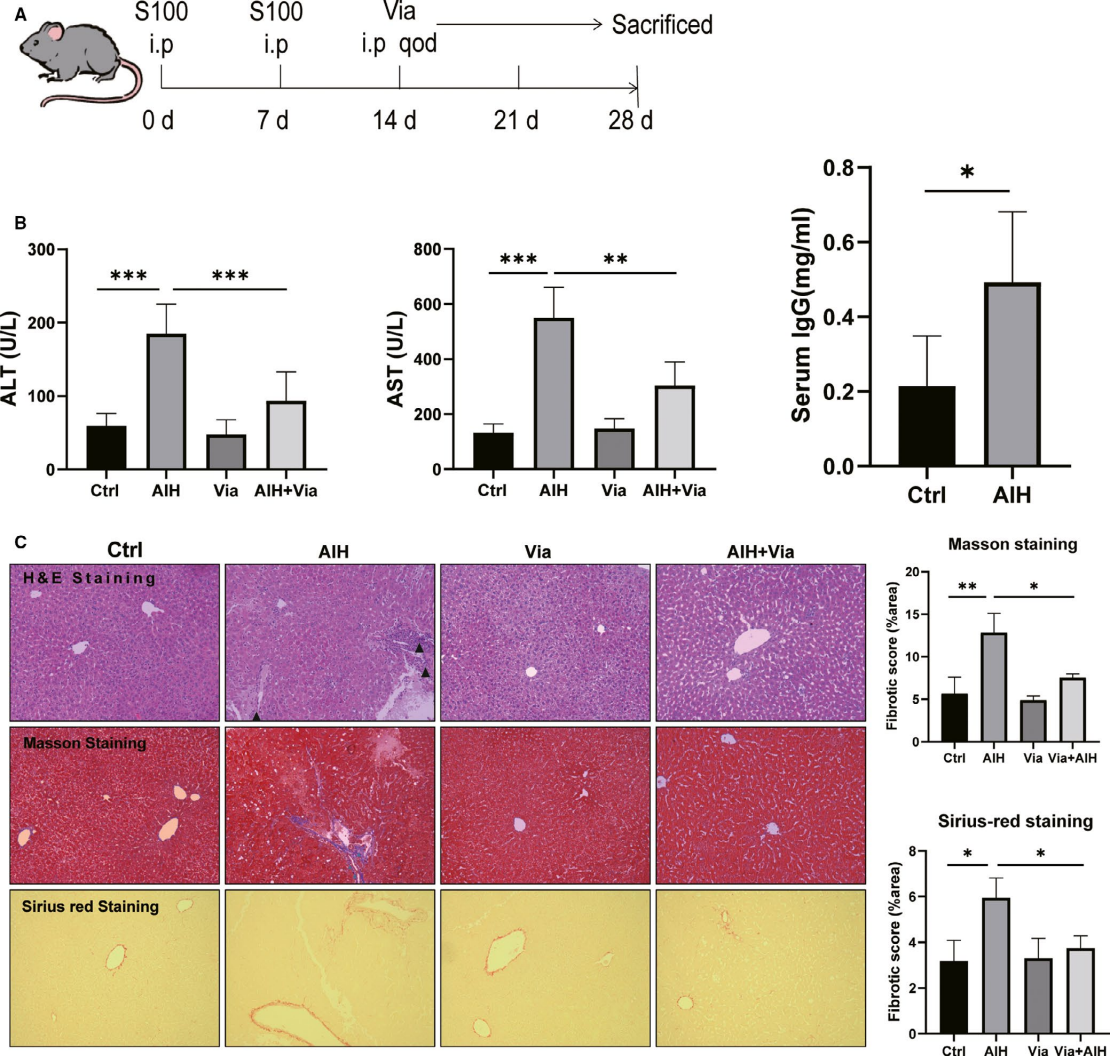
Vialinin A attenuates the inflammation and fibrosis of the liver in AIH mice. Panel A shows the protocol of establishment of autoimmune hepatitis in mice and Via injection. Panel B displays the serum levels of ALT and AST from each group, IgG from Ctrl and AIH group. Panel C shows the representative pictures of H&E, Masson and Sirius red staining of liver tissues. Black triangle highlights the lymphocytic infiltration (magnification, ×200). The right panels represent quantification of the fibrotic area from Masson staining and Sirius red staining. The data are presented as mean ± SED from 6 mice. ***P < .001

C57BL/6 male mice (6‐8 weeks of age) were purchased from shanghai Charles river. The Wenzhou Medical University Animal Policy and Welfare Committee approved all animal care and experimental procedures (Approval Document No. wydw2017‐0023), and all experiments were performed in accordance with the Health guidelines of the National Institutional (Guide for the care and use of laboratory animals). The animals were adapt to the laboratory for 2 weeks before initiation of the studies. Autoimmune hepatitis model in mice was generated by injection of freshly prepared S100 from mice. S100 antigen was prepared after perfusion of livers with normal saline and homogenizing of the livers. After centrifugation at 100 000*g* for 2 hours, the supernatants was S100 and the solution was concentrated to 0.5 mL using an Amicon Ultra‐15 filter (Millipore) and subsequently passed through a 90 cm CL‐6B Sepharose column (Solarbio) with the AKTA pure (GE Healthcare). There were three protein peaks collected from the column, the peak 1 and peak 3 components were better than the peak 2 as liver antigen. In this experiment, the concentration of the peak 3 solution is 0.5‐2.0 g/L. For immunization, with an equal volume of complete Freund's adjuvant (day 0) or incomplete Freund's adjuvant (day 7), the liver S100 antigen was emulsified. This mixture injected to mice intraperitoneally on day 0 and on day 7 again (Figure 1A). Because two mice died during the experiment. 12 established autoimmune hepatitis mice and 12 control mice were randomly selected half for treatment of Vialinin A. Vialinin A (5 mg/kg, dissolve in corn oil) was injected intraperitoneally once every two days starting from day 14 and on day 28, mice were narcotized with ixty microlitres of 6% ethal and sacrificed for the study.

### 2.3 Liver histopathology examination

Liver tissues were fixed in 4% paraformaldehyde and paraffin which collected from all mice. 5‐μm sections were prepared and next used to stain with H&E, Masson and Sirius red to evaluate the lymphocytic infiltration, fibrosis content and hepatocyte necrosis.

### 2.4 Human subjects blood specimens

The study included five independent patients who were clinically diagnosed as autoimmune hepatitis and five healthy volunteers. All subjects were not participants in any other clinical trials. The research was conducted according to The Code of Ethics of the World Medical Association (Declaration of Helsinki) and were approved by the institutional review board of hospital (ChiCTR‐OCS‐11001173). All subjects were written informed consents. The healthy volunteers were recruited. The AIH patients blood specimens were collected during hospitalization.

### 2.5 Cell culture and transfection

AML12 cells were maintained in DMEM/F‐12 with 10% FBS, which added 40 ng/mL Dexamethasone and 1% ITS at 37°C in 5% CO_2_. When cells were subcultures and grown to 70%‐80% confluence, cells were treated with Vialinin A (5 µmol/L) and LPS (5 µg/mL) for 12 hours. When cells were subcultures and grown to 30%‐40% confluence, transfected with non‐specific control siRNA and USP4 specific knockdown siRNA respectively with LipofectAMINE™ 2000 (Thermo Fisher) according to the manufacturer's procedure. Cell culture medium was changed to OPTI medium for 6 hours and then in DMEM/F12 with 10%FBS for 36 hours after transfection. Non‐specific control siRNA and siRNAs for USP4 were purchased from GenePharma. The sequences for non‐specific control siRNA and USP4 specific siRNA are listed in Table 1.

**TABLE 1:** The sequences for non‐specific control siRNA and USP4 specific siRNA

siRNA	Sense	Antisense
USP4	GCAAAUGGUGAUAGCACUATT	UAGUGCUAUCACCAUUUGCTT
Non‐specific control	UUCUCCGAACGUGUCACGUTT	ACGUGACACGUUCGGAGAATT

### 2.6 RT‐PCR

Total RNA was isolated from frozen liver tissue, culture cells and human mononuclear cells (consent was obtained for experimentation with human subjects. The privacy rights of human subjects must always be observed) by TRIzol reagent according to the manufacturer's protocol. Then, total RNA was used to compound cDNA using the Prime ScriptTM RT reagent kit (Takara) according to the manufacturer's protocol. Quantitative real‐time PCR was performed to measure the messenger RNA levels of Col‐1, α‐SMA, IL‐1b, IL‐6, TGF‐b1, FN14, USP4 using TB Green Premix Ex TaqTM II (Takrara) in ABI7500 Fast real‐time PCR system (Applied Biosystems). The number of cycles is set to 35. The expression of individual genes was normalized to the expression of GAPDH using $2^{‐\Delta C_{T}}$ method. The quantity of mRNA relative to a reference gene was calculated by the $2^{‐\Delta \Delta C_{T}}$ method. The primers’ sequences are listed in Table 2.

**TABLE 2:** The primer sequences of genes in real‐time qPCR assay

Gene	Species	Forward	Reverse	Melting temperature (°C)
USP4	Mouse	TCTGGCTTCTCTGCTTCGTA	GAGGGTTGTCTCGGTTGAT	81.5
Col I	Mouse	TGCCGTGACCTCAAGATGTG	CACAAGCGTGCTGTAGGTGA	79.5
α‐SMA	Mouse	GTGCTATGTCGCTCTGGACTTTGA	ATGAAAGATGGCTGGAAGAGGGTC	86.5
IL‐1b	Mouse	CCCAAGCAATACCCAAAGAA	GCTTGTGCTCTGCTTGTGAG	81.5
IL‐6	Mouse	GGCAAGCCTTCCAGTTAGTCTTCC	AGAGTAAGCGTCCAGAGGTCAGC	84
TGF‐b1	Mouse	CAACCCAGGTCCTTCCTAAA	‐GGAGAGCCCTGGATACCAAC	82.3
FN14	Mouse	GTGTTGGGATTCGGCTTGGT	GTCCATGCACTTGTCGAGGTC	81
USP4	Human	TTTCCTGGCCCAATAGACAAC	GGTAGGGACCAATACATAGTCCA	79

### 2.7 Immunoprecipitation and western blotting

Cell lysates were prepared from AML12 cells after homogenizing in Tissue Lysis Buffer. The Pierce BCA protein assay kit was used to estimate protein concentration according to the manufacturer's protocol. According to the manufacturer's protocol, whole‐cell lysates were pre‐cleared with 40 µL of Protein A + G agarose beads (beyotime) for 1 hour at 4°C. Lysates were then incubated with 2 mg of the desired antibody overnight at 4°C. Subsequently, incubation at 4°C with 20 µL of Protein A + G beads for a further 1 hour. The immunoprecipitated solution was washed five times with lysis buffer and maintained in 30 µL volume. After denaturomg wth isopyknic 2× loading buffer, they were separated by SDS‐PAGE and immunoblotted with the indicated antibodies. For visualization, Blots used PVDF membrane (Millipore) and immunoblot analysis detection system (Biorad). For reprobing, membranes were incubated in a stripping buffer (Beyotime, P0025N) for 5 minutes and washed twice before reprobing.

### 2.8 Quantification and statistical analysis

The data was analysed using GraphPad Prism 5 software. All experiments were repeated not less than three times. The statistical significance of two groups was evaluated by Student's *t*‐test. Between multiple groups, statistical significance was evaluated by one‐way ANOVA or two‐way ANOVA with Fisher's LSD test or Bonferroni test. Data were shown as mean ± SEM *P* < .05 was considered statistically significant.

## 3 RESULTS

### 3.1 Vialinin A attenuates inflammation and fibrosis in S100‐induced autoimmune hepatitis

**FIGURE 2:**
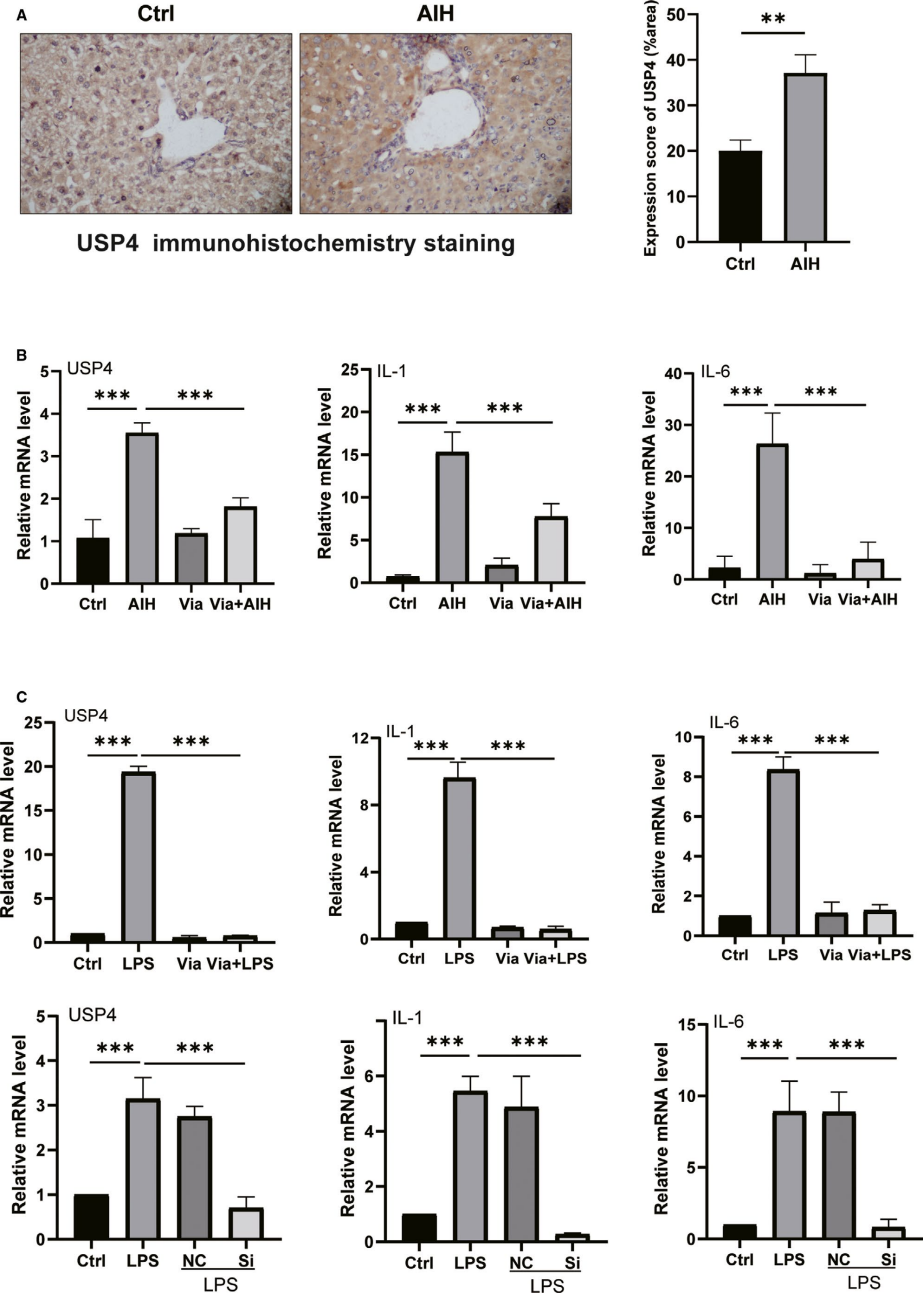
The expression of USP4 and the mRNA levels of inflammatory cytokines in the liver of AIH mice and AML12 cells. Panel A shows representative immunohistochemistry staining (magnification, ×400) of USP4 and the USP4 expression score in liver of normal mice and AIH model. Panel B displays the histogram of relative mRNA levels of USP4, IL‐1 and IL‐6 in the liver from each group. Panel C shows the histogram of relative mRNA level from AML12 cells treated with LPS plus or minus Vialinin A or specific inhibitory siRNA of UPS4. GAPDH used as loading control. The data are presented as mean ± SDM from 6 mice or 3 independent experiments of cells. **P < .01 and ***P < .001

**FIGURE 3:**
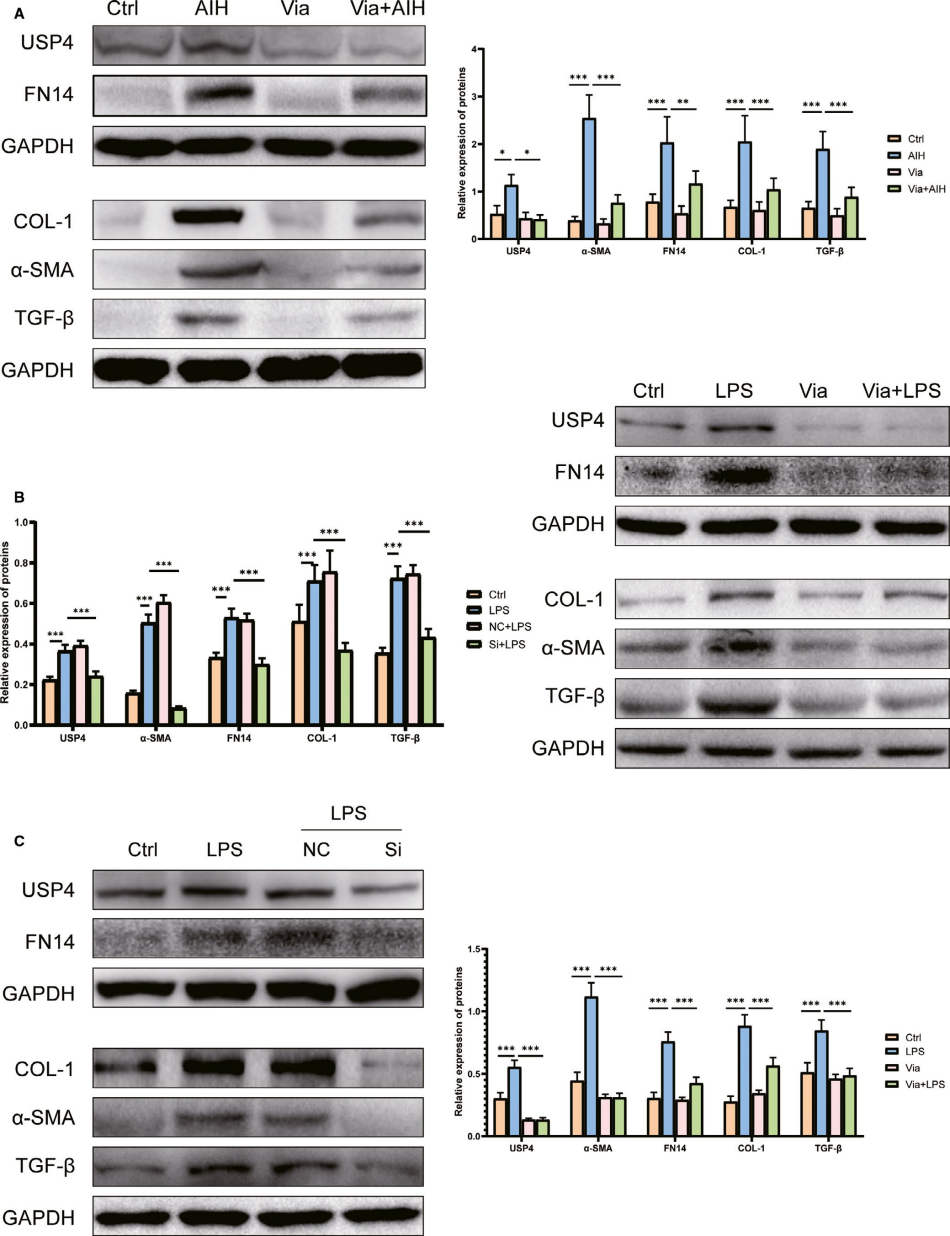
The protein levels of USP4 and fibrotic cytokines in mice and AML12 cells. Panel A shows the representative Western blot and histogram of proteins for USP4, COL‐1, α‐SMA, TGF‐β and FN14 in from each group. Panel B displays the typical Western blot and histogram of proteins of fibrotic cytokines from AML12 cells treated with LPS plus or minus Vialinin A. Panel C shows the typical Western blot and histogram of proteins different fibrotic cytokines proteins from AML12 cells treated with LPS specific inhibitory siRNA of UPS4. GAPDH used as loading control. The data represents mean ± SDM from 6 mice or 3 independent experiments from cells. *P < .05, **P < .01 and ***P < .001

The protocol of inducing autoimmune hepatitis with S100^7^, ^17^ was listed in Figure 1A. As shown in Figure 1, there were significant increases in ALT and AST values in AIH mice (Figure 1B). Moreover, in AIH mice, there were more necrotic areas, increased lymphocyte infiltration and disruption of liver structure (Figure 1C). USP4 level was significantly elevated in AIH mice (Figure 2A). In addition, Vialinin A treated mice showed normal transaminases, liver histology and USP4 level. Furthermore, when AIH mice were treated with Vialinin A, the USP4 level was reduced (Figures 2A and 3A). At the same time, Vialinin A also attenuated elevations of ALT and AST as well as liver histological changes (Figure 1B,C).

**FIGURE 4:**
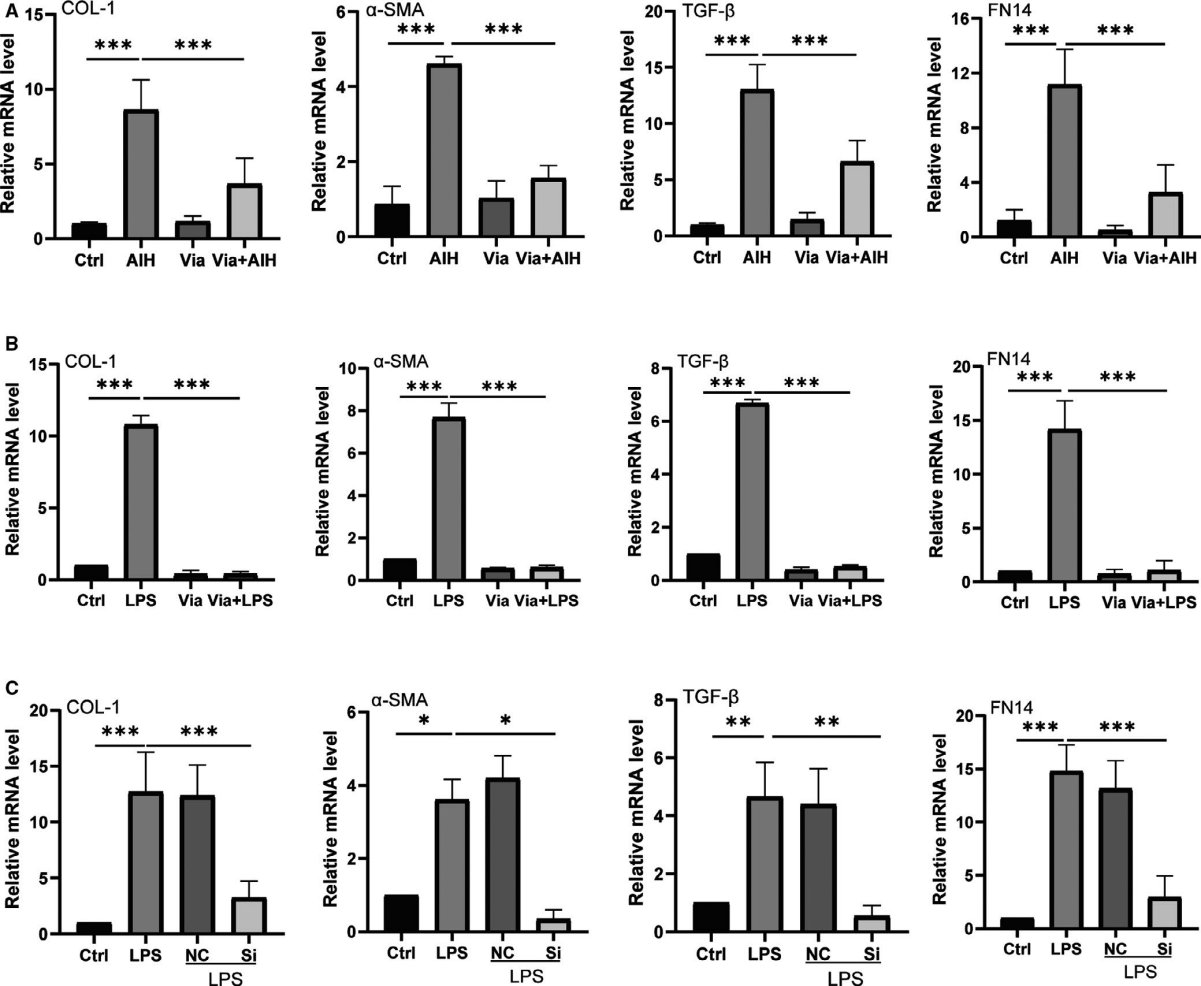
The mRNA levels of fibrosis markers from experimental mice and AML12 cells. Panel A displays relative mRNA levels of COL‐1, α‐SMA, TGF‐β and FN14 from each group. Panel B shows the relative mRNA levels of fibrosis markers from AML12 cells treated with LPS plus or minus Vialinin A. Panel C displays the same mRNA levels from AML12 cells treated with LPS alone or LPS with non‐specific or specific inhibitory siRNA of USP4. GAPDH used as loading control. The data are presented as mean ± SDM. *P < .05, **P < .01 and ***P < .001

Vialinin A could not only attenuate inflammation of S100‐induced autoimmune hepatitis, but also reduce liver fibrosis in AIH mice. As shown in Figure 1C, there were increased staining of Masson and Sirius red in the liver of AIH mice, however, Vialinin A treatment attenuated the degree of staining in the liver. Moreover, when liver cytokines (IL‐1, IL‐6), and fibrosis markers (COL‐1, α‐SMA, TGF‐β and FN14) were evaluated by Western blot (Figure 3A) and real‐time RT‐PCR (Figures 2A and 4A), there were significant increases in these markers at both protein and mRNA levels in AIH mice. However, Vialinin A treatment significantly attenuated the elevation of these markers in AIH mice but could not be able to bring them back to the level of normal mice.

### 3.2 UPS4 involved in LPS‐induced inflammation and fibrosis in AML12 cells

**FIGURE 5:**
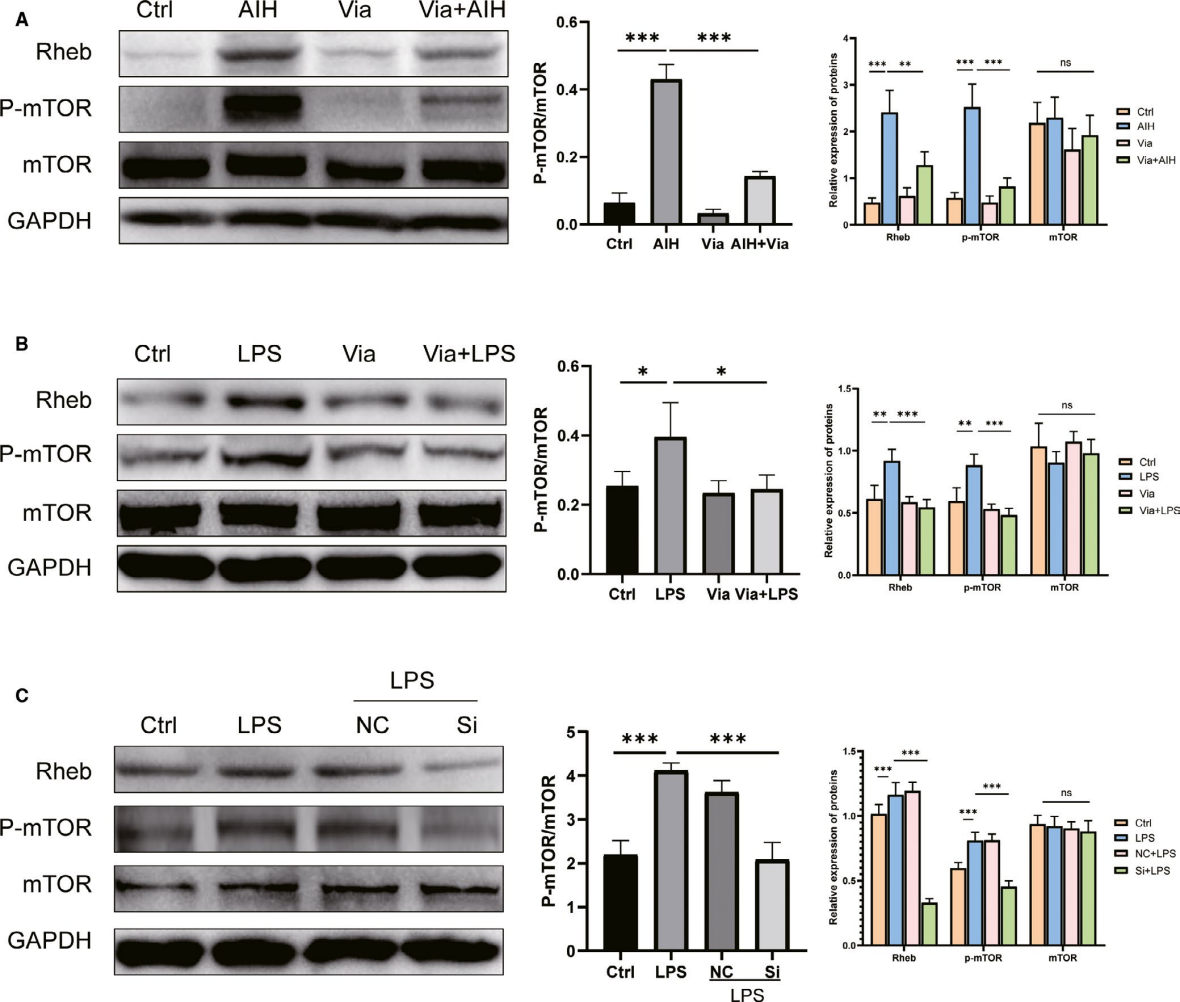
Alternation of Rheb/mTOR signalling in experimental mice and AML12 cells. Panel A shows representative Western blot pictures on the right and histigram of the band density on the right of Rheb, p‐mTOR, mTOR in the liver of each experimental group while panel B shows the same from AML12 cells treated with LPS plus or minus Vialinin A and panel C display the same form AML12 cells treated with LPS alone or LPS with non‐specific and specific inhibitory siRNA of UPS4. GAPDH used as loading control. The middle column shows the ratio of phosphorylated mTOR to mTOR. The data are presented as mean ± SDM. *P < .05, **P < .01 and ***P < .001

In order to better understand the mechanisms of UPS4 in liver inflammation and fibrosis, AML12 cells were employed and treated with LPS to induce cell injury. As shown in Figure 3B, LPS treatment induced a significant increase in UPS4 and other liver fibrosis markers such as COL‐1, α‐SMA, TGF‐β and FN14. Moreover, there were also increases in Rheb and phosphorylation mTOR especially the ratio of phosphorylated mTOR to mTOR in LPS‐treated cells (Figure 5B,C). Accordingly, the mRNA levels of USP4 and other markers (IL‐1, IL‐6, COL‐1, α‐SMA, TGF‐β and FN14) were also elevated by LPS in AML12 cells (Figures 2B and 4B). However, Vialinin A treatment significantly attenuated the elevation of these markers in AML12 cells treated with LPS. Furthermore, when employing non‐specific and specific inhibitory siRNA of USP4 to incubated with LPS‐treated AML12 cells, specific inhibitory siRNA of UPS4 significantly reduced LPS‐induced USP4 level at both protein (Figure 3C) and mRNA levels (Figure 2C). At the same time, other liver inflammatory (Figure 2C) and fibrosis markers (Figures 3C and 4C) were also reduced in AML12 cells treated with both LPS and USP4 inhibitory siRNA.

### 3.3 Vialinin A regulates the Rheb/mTOR signalling in S100‐induced liver injury and LPS‐induced cell injury

Whether Vialinin A can regulate Rheb/mTOR sginaling as USP4, AIH mice and AML12 cells were employed. As shown in Figure 5, in the liver of AIH mice, there were significant elevations of both Rheb and phosphorylated mTOR especially the ratio of phosphorylated mTOR to mTOR (Figure 5A). Vialinin A could significantly reduce the levels of both Rheb and phosphorylated mTOR. In addition, LPS also significantly increased the levels of Rheb and phosphorylated mTOR. Vialinin A also reduced both levels of Rheb and phosphorylated mTOR especially the ratio of phosphorylated mTOR to mTOR that were induced by LPS (Figure 5B). Signalling molecules were also reduced in AML12 cells treated with both LPS and specific inhibitory siRNA of USP4 (Figure 5C).

### 3.4 Mechanism of Vialinin A in attenuation of liver injury in AIH mice is mediated by regulation of Rheb/mTOR signalling through USP4

**FIGURE 6:**
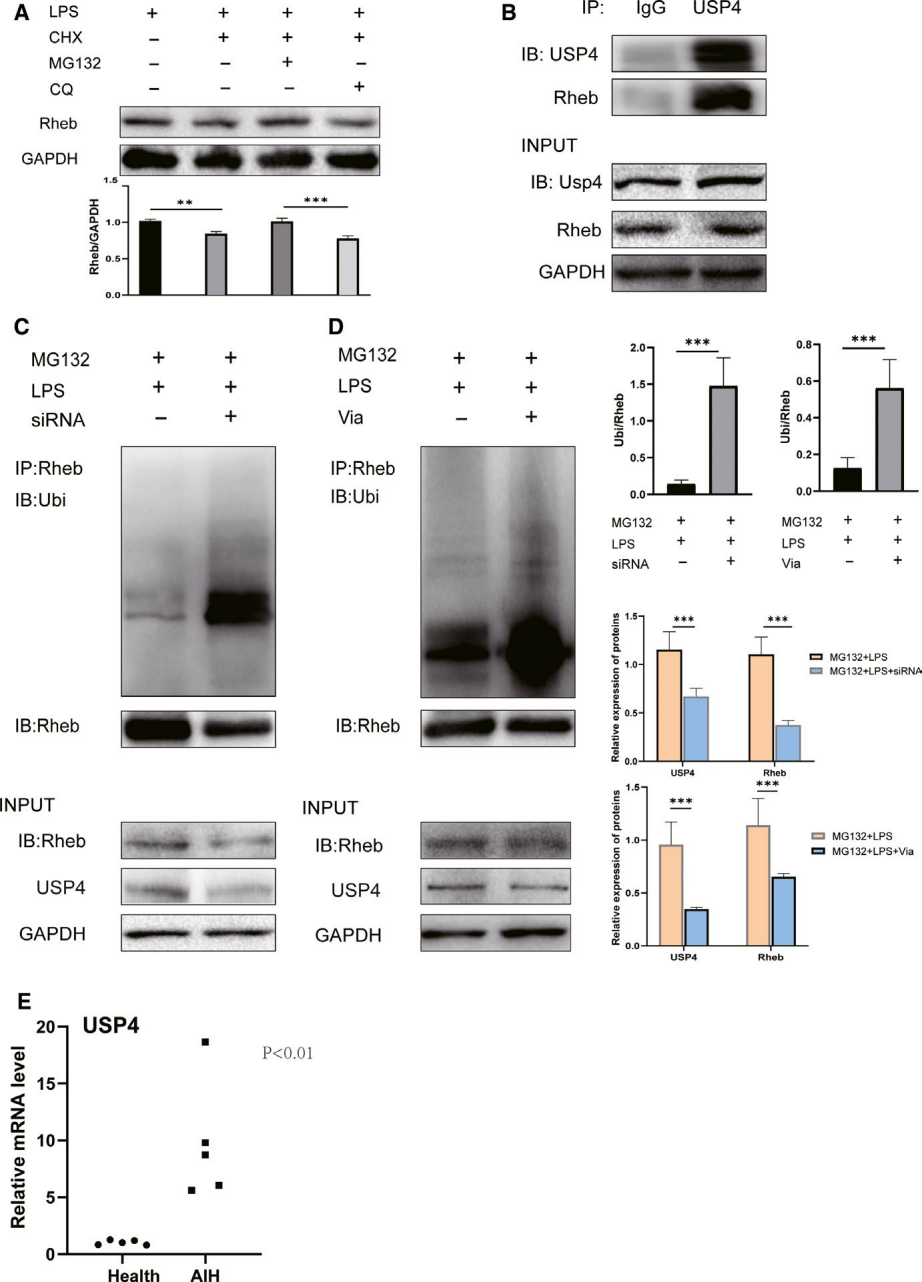
Immunoprecipitation experiments shows USP4 and Vialinini A involved in regulation of Rheb deubiquitination. Panel A shows the Rheb protein level in AML12 cells treated with 5 µmol/L LPS for 24 h and further treated with different protein inhibitors individually or in combination for 6 h of CHX (100 mg/mL), lysosomal inhibitor chloroquine (50 µmol/L) with and proteasome inhibitor MG132 (5 µmol/L). Panel B shows the immunoprecipated with USP4 and immunoblot with USP4 and Rheb with immunoprecipitaed proteins. Panel C displays the immunoprecipation experiments with proteasome inhibitor MG132 and LPS as well as with and without specific inhibitory siRNA of USP4. Protein was immunoprecipitated with antibody of Rheb and immunoblotted with Ubiquitin antibody. Panel d shows the same immunoprecipitation experiments as in panel C except with Vialinin A instead of specific inhibitory siRNA of USP4. The right Panel displays the histogram of (C) and (D). Panel e shows USP4 mRNA level from healthy and AIH patients. The data are presented as mean ± SDM from 6 mice of 3 independent experiments of cells. **P < .01 and ***P < .001

Since the role of Vialinin A in LPS‐induced AML12 cell injury is similar to its function in AIH liver injury (Figure 5), AML12 cells were employed to identify the mechanism of Vialinin A for attenuation of liver injury in AIH by using immunoprecipitation (IP) experiments. As shown in Figure 5, when AML12 cells were incubated with LPS or LPS with other inhibitor, Rheb level was reduced when cells were incubated with cycloheximide (CHX). Moreover, when cells were further incubated with combination CHX and a lysosomal inhibitor (chloroquine—CQ) or a proteasomal inhibitor (MG132), only MG132 prevented CHX‐induced reduction of Rheb suggesting Rheb degradation is mediated by proteasome pathway. Furthermore, interaction of USP4 and Rheb was examined by immunoprecipitation assay (Figure 6B). To identify the relationship between USP4 and Rheb degradation, AML12 cells treated with MG132 and LPS plus or minus inhibitory siRNA of USP4. Inhibition of USP4 significantly produced ubiquitination level of Rheb in immunoprecipitated solution, although same amount of Rheb and USP4 proteins were loaded to immunoprecipitation solution (Figure 6C). The similar result of Rheb immunoprecipitation was observed in cells treated with MG132 and LPS plus or minus Vialinin A. Vialinin A treated cells showed increased level of immunoprecipitated Rheb with the same amount of protein loading (Figure 6D).

### 3.5 The expression of USP4 in the blood of AIH patients is higher

To further elucidate the regulatory roles of USP4 in AIH patients, we examined the expression of USP4 in several AIH patients blood circulating mononuclear cells. PCR results showed that level of USP4 was significantly up‐regulated in AIH patients compared with matched healthy volunteers (Figure 6E). Furthermore, the result requires confirmation in a larger cohort for more definitive findings.

## 4 DISCUSSION

Vialinin A is an extract from mushroom has been shown to regulate USP4 in vitro. However, its role in regulation of USP4 and others is still not clear, especially in autoimmune hepatitis. In the current study, we demonstrated the impact of Vialinin A in inflammation and fibrosis of an in vivo model of autoimmune hepatitis. It clearly indicates that Vialinin A can reduce USP4 level in the liver of autoimmune hepatitis, which contributes to reduction of inflammation and fibrosis in the liver. This is partially consistent with the observation that Vialinin A can inhibit TNF‐a expression,^18^, ^19^ since TNF‐a is a potent inflammatory cytokine. However, Vialinin A is a more potent inhibitor of ubiquitin‐specific peptidase.

Ubiquitin‐specific proteases as thiol proteases share a structurally conserved catalytic domain with a catalytic residues. Vialinin A strongly inhibited the enzymatic activities of USP5 and USP4 through inhibiting catalytic residues, but no significant inhibition was observed in other DUBs.^16^ Vialinin A has been used as a specific inhibitor of USP4 in vitro experience commonly,^12^, ^15^ but is seldom used as inhibitor of USP5 in previous study.

Ubiquitination generates a targeting signal that can be used to alter the properties or localization of the ubiquitinated protein and delivered ubiquitinated proteins to the proteasome, a large compartmentalized multi‐catalytic protease that is responsible for much of the regulated proteolysis in cells.^17^ However, USP4 as an deubiquitinating enzymes reverses the ubiquitination. With the regulatory role of deubiquitination in inflammation reaction is highly valued, a key role for the deubiquitinase USP4 has been reported in some liver disease. However, the conclusion of the role of USP4 play in liver disease is converse. As some reported, USP4 deficiency intensifies the inflammation in the liver of hepatic I/R injury^8^ and NAFLD.^13^, ^20^ While other studies suggest that USP4 over‐expression promotes the progress of fibrosis in the liver,^11^ as well as the metastasis of hepatocellular carcinoma.^9^, ^21^, ^22^ Our present study clearly demonstrated that inhibiting USP4 expression alleviates the fibrosis progress in autoimmune hepatitis. Moreover, we examined the expression of USP4 in AIH patients, which was much higher in healthy person. A recent studies also found that USP4 is overexpressed in human HCC.^21^, ^22^

As previous studies,^23^, ^24^, ^25^, ^26^ mTOR signal pathway inhibition suppressed liver fibrosis in other liver disease models. A recent study confirmed that USP4 plays a stimulative role in Rheb signal.^15^ Therefore, we further explored the role of Rheb/mTOR pathway in the liver of autoimmune hepatitis. The existence of USP4 decreased the degradation of Rheb through reversing the ubiquitination. Then, the Rheb/mTOR pathway was stimulated. In the current study, we discovered that the activation of Rheb/mTOR pathway drives the tendency to liver fibrosis, which is consistent with findings in other liver diseases.

The ubiquitin‐proteasome system (UPS) has developed as a therapeutic target for the treatment of cancers.^27^ In this study, the finding that USP4 has a crucial regulatory role in autoimmune hepatitis fibrosis may provide new clues for AIH treatment and Vialinin A may provides a new therapeutic drug for AIH patients.

## CONFLICT OF INTEREST

All authors have no potential conflict of interest to declare. All authors read and approved of the final version of the manuscript. All authors have read the journal's policy on conflicts of interest. All authors have read the journal's authorship agreement.

## AUTHOR CONTRIBUTIONS

**Jie Xu:** Conceptualization (lead); Data curation (lead); Formal analysis (lead); Methodology (lead); Resources (lead); Software (lead); Validation (equal); Writing‐original draft (lead). **Chen Dazhi:** Conceptualization (equal); Data curation (equal); Formal analysis (equal); Methodology (equal); Validation (equal); Writing‐review & editing (equal). **Lanling Jin:** Conceptualization (equal); Resources (equal); Software (equal). **Zhengkang Chen:** Resources (equal); Software (equal). **Yulu Tu:** Conceptualization (supporting); Data curation (supporting). **Xiaozhe Huang:** Resources (supporting); Software (supporting). **Feiben Xue:** Resources (supporting); Validation (supporting). **Jialu Xu:** Data curation (supporting); Methodology (supporting). **Mingzhuan Chen:** Software (supporting). **Wang Xiaodong:** Writing‐review & editing (supporting). **Yongping Chen:** Funding acquisition (lead); Project administration (lead); Writing‐review & editing (lead).

## Data Availability

The data used to support the findings of this study are included within the article.
